# A critical review of hot executive functioning in youth attention-deficit/hyperactivity disorder: Methodological limitations, conceptual considerations, and future directions

**DOI:** 10.1017/S0954579422001432

**Published:** 2023-02-03

**Authors:** Jessica N. Smith, Morgan L. Jusko, Whitney D. Fosco, Erica D. Musser, Joseph S. Raiker

**Affiliations:** 1 Florida International University (FIU), USA; 2 Pennsylvania State University Hershey Medical Center, USA; 3 FIU Center for Children and Families, USA; 4 FIU Embrace, USA

**Keywords:** attention-deficit/hyperactivity disorder, disruptive behavior problems, hot executive functioning, measurement, task design

## Abstract

Hot executive functioning (EF) – EF under emotionally or motivationally salient conditions – is a putative etiology of attention-deficit/hyperactivity disorder (ADHD), disruptive behavior problems (DBPs), and their related impairments. Despite two decades of research, the present study is the first review of the construct in youth ADHD, with a particular focus on the role of task design, age, and DBPs, as well as relevant conceptual and methodological considerations. While certain hot EF tasks have been investigated extensively (e.g., choice impulsivity), substantial inconsistency in measurement of the broader construct remains, severely limiting conclusions. Future research should a) consider the extent to which various hot EF tasks relate to one another, a higher order factor, and other related constructs; b) further investigate task design, particularly the elicitation of emotion or motivation and its anticipated effect on EF; and c) incorporate multiple levels of analysis to validate similarities and differences among tasks with regard to the affective experiences and cognitive demands they elicit. With improved measurement and conceptual clarity, hot EF has potential to advance the literature on etiological pathways to ADHD, DBPs and associated impairments and, more broadly, may represent a useful tool for understanding the influence of emotion and motivation on cognition.

## Introduction

Executive functioning (EF) refers to the largely interdependent processes necessary for goal-directed behavior, with various models isolating the cognitive processes of interest (Goldstein et al., 2014). For example, working memory, set shifting, and inhibition have been identified as three primary EFs necessary for successful task execution (Miyake et al., 2000). Historically, the term has invoked a primarily cognitive lens focused on decontextualized, abstract problem solving (Peterson & Welsh, 2014). Despite this primarily cognitive framework for understanding these “cool” EFs, the term “hot executive functioning” (i.e., hot EF) emerged two decades ago (Zelazo & Müller, 2002) to characterize how EFs operate within the context of motivationally or emotionally salient situations (Bechara, 2004; Bell & Wolfe, 2004; Somerville & Casey, 2010). Considerations of EFs in these contexts enhances external validity given the complex real-world scenarios in which EFs are invoked to problem solve and set goals (Castellanos et al., 2006; Peterson & Welsh, 2014). To this end, the hot EF construct is likely to serve as a helpful tool for advancing our understanding of psychopathology. For example, the distinction between cool and hot EFs has been discussed as reflecting distinct etiological processes that give rise to various disorders (Séguin et al, 2007; Zelazo, 2020). Further, funding agencies such as the National Institutes of Health increasingly acknowledge the complex interdependency among cognition, emotion, and motivation (Cuthbert, 2014; Lilienfeld & Treadway, 2016), resulting in calls to consider these constructs synergistically (Tsermentseli & Poland, 2016; Welsh & Peterson, 2014).

Attention-deficit/hyperactivity disorder (ADHD) has garnered particular attention with respect to both cool EFs (e.g., working memory; inhibition) and hot EFs. Youth with ADHD evidence well-documented impairments in cool EFs at a group level (Pievsky & McGrath, 2018), and poor cool EF is one of the most heavily implicated etiologies of ADHD (Crippa et al., 2015). Notably, however, not all children with ADHD exhibit the same impairments in these areas, and some exhibit no impairment at all (e.g., Fair et al., 2012; Kofler et al., 2019). Given this oft-replicated heterogeneity in cool EF, proponents of the dual pathway model have suggested there may be distinct executive and motivational dysfunction pathways that contribute to the sequela of ADHD (Pauli-Pott et al., 2019; Sonuga-Barke, 2002). In addition to within-disorder heterogeneity, hot EF may be useful for understanding ADHD’s high rate of comorbidity with disruptive behavior disorders (Gau et al., 2010). While cool EFs are primarily associated with ADHD, particularly inattention, hot EFs tend to be associated with disruptive behavior problems (DBPs) such as oppositional defiant disorder (ODD) or conduct disorder (CD; Hobson et al., 2011; Noordermeer et al., 2020; Pauli-Pott et al., 2019; Thorell, 2007). This is important given that DBPs are often associated with more severe adverse outcomes (Allen et al., 2019; Connor & Doerfler, 2008; Loeber et al., 2000). Relatedly, preliminary work has demonstrated that individuals with deficits in cool EF experience different impairments and outcomes relative to those with deficits in hot EF (Skogli et al., 2017; Thorell, 2007). Collectively, hot EF has significant implications for understanding the etiology of ADHD, its co-occurrence with DBPs, and associated impairments.

## Present review

### Scope

Given the potential importance of hot EF for understanding ADHD, it is unsurprising that interest in the construct persists (and, in fact, has substantially increased). For example, in our cursory search for the terms *hot executive functioning* and *ADHD* in Google Scholar, over 3,700 results were returned between the years 2000 and 2010, whereas nearly 13,000 results were returned between the years 2011 and 2021. There have been recent reviews of “hot EF” in typically developing (TD) preschoolers, which have generally concluded that the term lacks conceptual clarity and that there has been limited methodological consistency employed across studies investigating hot EF (Garon, 2016; Welsh & Peterson, 2014). However, there is no review of hot EF for youth (i.e., children and adolescents) with ADHD. Before delineating the foci of the present review, specifying the scope of hot EF to be reviewed is necessary.

**Figure 1.**
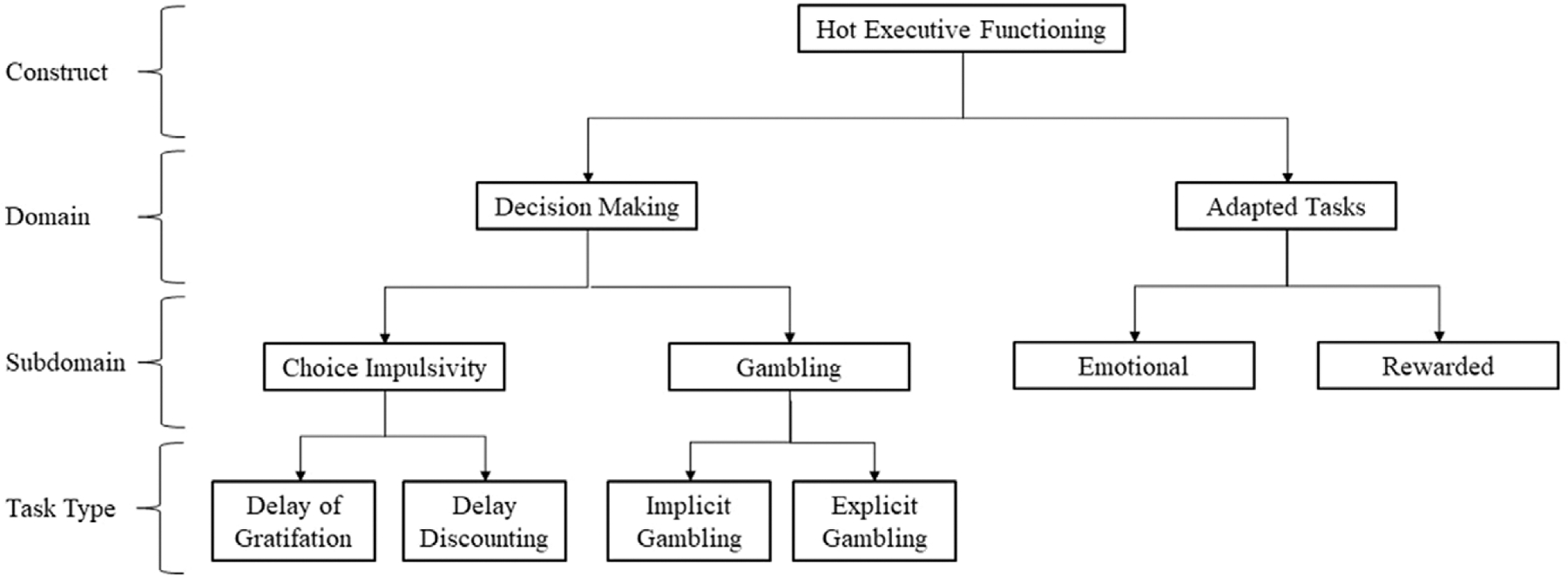
Conceptual diagram of hot executive functioning domains, subdomains, and task types reviewed.

The present review prioritized two task domains in hopes of narrowing the scope and providing a more concrete synthesis. This includes tasks most frequently utilized in studies of ADHD purporting to measure “hot EF” as well as tasks designed to assess “EFs under emotionally or motivationally salient contexts” (Zelazo & Müller, 2002). The most common tasks used to assess for hot EF in ADHD are decision-making (DM) tasks, including choice impulsivity and gambling (e.g., Antonini et al., 2015; Dolan & Lennox, 2013; Hobson et al., 2011; Kerr & Zelazo, 2004; O’Toole et al., 2018; Poland et al., 2016; Poon, 2018; Prencipe et al., 2011; Skogli et al., 2014, 2017; Yang et al., 2011). These tasks are also utilized extensively in ADHD research without use of the term “hot EF,” as evidenced by many available reviews and meta-analyses on DM, choice impulsivity, or gambling (e.g., Dekkers et al., 2016, 2021; Marx et al., 2018; Patros et al., 2016; Pauli-Pott & Becker, 2015; Roberts et al., 2021; Schulze et al., 2021). These extant meta-analyses and reviews will be included in order to synthesize all available evidence on these tasks in youth ADHD as they pertain to hot EF. A second, albeit less common, approach to assessing hot EF involves modifying a traditional cool EF task by integrating a “hot” (i.e., emotional or motivational) element (e.g., Hobson et al., 2011; Ma et al., 2016; Rubia et al., 2009a; Van Cauwenberge et al., 2015; Zhu et al., 2021), which we will call an “adapted task” (AT). Adapted tasks (ATs) can be further specified as emotional (e.g., emotional working memory) or rewarded (e.g., rewarded continuous performance task [CPT]). While ATs have been utilized less frequently, they are included in this review as these modifications are consistent with a conceptualization of hot EF that reflects how EF processes operate within motivationally or emotionally salient contexts. See Figure 1 for an overview of the hot EF task domains reviewed herein.

It is important to note that, despite the popularity of “hot EF” in the ADHD literature, there is no clear consensus regarding what constitutes a hot EF task. Thus, the present review may not capture all possible measures of the construct. While “hot EF” has been used broadly to refer to EF in emotionally or motivationally salient situations, other work has put forth more precise definitions and uses. For example, the specific (and unique from cool EF) top-down processes needed for emotional and motivational contexts (Zelazo & Carlson, 2012); flexible reappraisal used for evaluating whether to approach or avoid a stimulus, which is utilized in delay of gratification, delay discounting, and gambling (Zelazo, 2020); as well as hot EF’s role in other processes like emotion regulation and theory of mind (Zelazo, 2020). Importantly, Zelazo clarifies that research on “hot EF” is independent from research on a “hot-cool systems” framework which focuses on bottom-up emotional influences and does not focus on EF at all (Metcalfe & Mischel, 1999; Zelazo & Carlson, 2012). Importantly, however, the “hot” moniker is often used without specification of what use of “hot” EF is being referenced throughout the literature. The present review uses hot EF in reference to tasks consistent with the Zelazo and Müller (2002) conceptualization (i.e., ATs) and the term’s popular usage (i.e., DM).

Relatedly, because the terms “reward sensitivity” and “motivation” are often used interchangeably in the ADHD literature (Smith & Langberg, 2018), tasks of reinforcement learning, reward and punishment sensitivity, and hot EF often overlap, despite presumably assessing different constructs. Indeed, while many studies utilize tasks specifically designed to assess reinforcement learning or reward/punishment sensitivity, there are also studies focusing on “reinforcement” that utilize gambling (e.g., Groen et al., 2013; Humphreys & Lee, 2011; Masunami et al., 2009; Matthys et al., 2004; Tenenbaum et al., 2018) and choice impulsivity (e.g., Richards et al., 2016; Van Hulst et al., 2015) tasks. Importantly, however, the dependent variable in reinforcement learning studies is not the change in EF performance itself, but rather, what can be inferred about a participant’s reward/punishment sensitivity, often focusing primarily on neurobiology or physiology rather than behavioral performance (e.g., Chevrier et al., 2019; Luman et al., 2010; Van Meel et al., 2005). Thus, such studies will not be included in this review. Lastly, while the present review focuses on youth ADHD, there is a large literature base on “hot EF” in TD preschoolers, which contributes to the diversity of tasks used under the “hot” moniker as they often incorporate a variety of interactive research assistant-administered delay tasks (e.g., Brock et al., 2009).

### Aims

The present critical review consists of two parts: First is a review of the available evidence, and second is a discussion of conceptual and methodological considerations. In the review of literature, we will consider 1) the extent to which task design is related to variability in effect size magnitudes; 2) the extent to which age is related to effect sizes; and 3) the role of DBPs that frequently co-occur with ADHD and whether they influence effect sizes. Task design was identified as an important focus given the methodological inconsistency highlighted by past reviews of hot EF in other populations (e.g., Welsh & Peterson, 2014). Age was chosen as an important focus given how little is known about hot EF in children and adolescents with ADHD, despite several reviews of the construct in preschoolers. Lastly, DBPs were chosen as a focus given theoretical postulations and emerging empirical findings that hot EF deficits in ADHD may be related to these symptoms (Hobson et al., 2011; Noordermeer et al., 2020; Pauli-Pott et al., 2019; Thorell, 2007). The phrase “DBPs” is used to refer broadly to ODD and/or CD due to variability across studies. See Supplemental Table 1 for a summary of findings discussed in the literature review. The discussion of conceptual and methodological considerations will 1) summarize what conclusions can be drawn regarding hot EF in ADHD; 2) discuss the similarities and differences among hot EF tasks; 3) consider issues related to construct validity, such as the elicitation of emotion and motivation and how to measure their effect on EF; 4) discuss the distinguishability of hot EF and various related constructs; 5) describe the utility of an approach that incorporates multiple levels of analysis; and 6) highlight limitations of the present review and future directions.

### Method

Regarding our methodology for the literature review, we utilized Google Scholar to locate articles with no date range limitations. The review of DM findings was largely dependent upon numerous extant meta-analyses and reviews, except for individual studies that tested an area of interest (i.e., task design, age, DBPs). To locate these, searches for *decision making, choice impulsivity, delay discounting, delay of gratification, and gambling crossed with ADHD and attention-deficit/hyperactivity disorder* were conducted. The review of ATs was largely reliant upon individual studies, except for two meta-analyses focused on the effects of reward on inhibition (Burton et al., 2021; Ma et al., 2016). To identify ATs, searches for *hot, emotional, motivational, or rewarded* crossed with a list of specific tasks (e.g., stop signal, go/no-go) as well as cognitive constructs (e.g., *inhibition, working memory*) were conducted, and resultant studies were forwards and backwards searched. In all searches, we sought to identify studies of children and adolescents where ADHD was the focus and where DM tasks or ATs were administered and behavioral results were described. If a study discussed both youth and adults, the results on youth were discussed as the primary focus. Authors aimed to objectively review the evidence in this section, and reserve discussion and commentary for the conceptual and methodological limitations section.

Importantly, we opted not to conduct a systematic review or meta-analysis for several reasons. A search for “hot EF” terms would not yield a consistent set of tasks and would simultaneously exclude tasks that are popularly considered to be hot EF that are frequently used without the “hot EF” label (e.g., the literature on choice impulsivity). Thus, we decided to focus our search on the tasks commonly used to assess hot EF (i.e., DM) and tasks that assess for hot EF by definition (i.e., ATs) rather than a search for “hot EF” itself. However, the DM literature already has been exhaustively reviewed, and simultaneously, the literature on ATs is not yet ripe for meta-analysis as the term is not an established one, the literature is rather sparse, and the extant studies are quite varied in their methods (see Supplemental Table 1). Instead, we opted to provide an overview of numerous extant reviews and meta-analyses of DM and a summary of identified studies using what we have determined to be ATs together in light of the hot EF construct. Consistent with the definitions of various types of reviews outlined by Grant and Booth (2009), we feel that this study meets their description of a *critical review*. Namely, Grant and Booth (2009) describe a *critical review* as one that involves extensive research of the literature and critical evaluation of its quality in order to take stock and provide a launch pad for future theoretical and empirical work. Collectively, our goal is to provide a broad perspective of the state of the hot EF literature in youth ADHD, to elucidate specific limitations in this literature, and to provide future directions that will foster clarity.

## Review of literature

### Decision-making tasks

Decision-making (DM) tasks assess the extent to which a participant makes suboptimal or risky choices in situations involving reward and can be further subdivided into choice impulsivity and gambling tasks (e.g., Dekkers et al., 2021; Roberts et al., 2021; Schulze et al., 2021). Furthermore, choice impulsivity tasks can be further delineated into delay of gratification (DG) and delay discounting (DD) tasks (Patros et al., 2016). While both types of choice impulsivity tasks require that participants choose between small, immediate reinforcers and larger, delayed reinforcers (Patros et al., 2016), they differ in how they estimate the dependent variable. Specifically, DG tasks typically present two choices across multiple reinforcement schedules that remain fixed across trials, and the dependent variable is how many choices were made for the immediate versus delayed reward. More choices for smaller, but more immediate reinforcement is considered reflective of an impulsive response style (Patros et al., 2016). DD tasks incorporate dynamic reinforcement schedules that estimate an indifference point, or the point at which a participant has an equal probability of choosing either the immediate or delayed reinforcer (i.e., when the subjective value of a smaller, immediate reward is considered to be equivalent to a larger reward at a particular delay; Patros et al., 2016; Wilson et al., 2011). Lower indifference points are reflective of a more impulsive response style. A second type of DM task are gambling tasks, during which participants choose between options that vary with respect to the odds of receiving rewards or penalties (Groen et al., 2013). Gambling tasks can be further subdivided into explicit or implicit. This distinction refers to the presence or absence of information provided to the participant regarding relevant probabilities (i.e., the risks and benefits of each of the available options in terms of obtaining a certain reward), respectively (Groen et al., 2013). For example, in the implicit Iowa Gambling Task, participants make 100 choices from four decks of cards, some of which lead to monetary gains or losses. While two decks are “risky” and disadvantageous and two decks are “safe” and advantageous, participants are not informed of this, and only have the opportunity to learn through the consequences of their successive choices (Bechara et al., 1994). Alternatively, in the explicit Cambridge Gambling Task, 10 red and blue boxes are presented on the screen in various ratios (e.g., 9:1, 7:3) and participants are instructed to guess the color of the box which contains the reward (Rogers et al., 1999).

Meta-analytic work reveals that, overall, DM tasks demonstrate moderate magnitude effect sizes (such that ADHD youth are more impaired in DM than their TD peers). Specifically, choice impulsivity tasks, on average, demonstrate moderate magnitude effect sizes in youth with ADHD relative to TD children (DG SMD = .36, DD SMD = .43, Marx 2018; *g* = .47, Patros et al., 2016; DD and DG *d* = .63, Pauli-Pott & Becker, 2015), though with substantial variability across studies (SMD = −0.13 to 0.87, Marx et al., 2018; *g* = .22 to 1.04, Patros et al., 2016; *d* = 0.17 to 1.40, Pauli-Pott & Becker, 2015).^1^ Similarly, gambling tasks demonstrate small-to-moderate effect sizes in ADHD compared to TD children with significant variability (SMD = .01 to 2.33, average = .36; Dekkers et al., 2016). Given the heterogeneity in effect sizes across tasks and constructs, we further consider the role of task selection, age, and DBPs as they relate to differences in effect size estimates.

#### Decision-making: task design.

Choice impulsivity tasks can involve real rewards or hypothetical rewards. Although DG tasks often involve real rewards and DD tasks often involve hypothetical ones, the magnitude of effect sizes are generally similar across these tasks. Specifically, Patros et al. (2016) found that DG studies resulted in an average Hedge’s g of .47, whereas DD tasks resulted in an average Hedge’s *g* of .50. Marx et al. (2018) also found similar effect sizes such that simple choice paradigms (i.e., DG tasks) produced an SMD of .36, while temporal discounting (i.e., DD) tasks produced an SMD of .43. Importantly, however, follow-up moderation analyses showed that the anticipation of real rewards during DG tasks halved choice impulsivity in ADHD youth (Marx et al., 2018), suggesting that the use of real rewards may be important and that effect size differences in meta-analyses were dampened by the use of both real and hypothetical rewards across DG tasks. Just as Marx et al. (2018) examined DG tasks with and without real rewards, future work should examine DD tasks with and without real rewards. Relatedly, the magnitude of real rewards has been investigated and was not found to impact effect size magnitudes when comparing ADHD and TD youth (Scheres et al., 2010). However, participants could only earn up to 10 cents per trial with a maximum total gain of 6–8 dollars; It is possible that the use of larger or more salient rewards may produce different findings.

Another consideration is whether *delays* are experienced or hypothetical in choice impulsivity tasks. DG tasks often have participants experience the chosen delay (in the seconds range), while the delays in DD are longer, but hypothetical (e.g., days, months, or years; Marx et al., 2018). The role of real versus hypothetical delays has not been examined directly to the authors’ knowledge. However, the impact of total session length on preference for smaller, more immediate rewards has been investigated, and was not found to influence effect size differences between ADHD and TD youth (Scheres et al., 2006, 2010). Future work examining the effect of real and hypothetical delays should do so for both DD and DG tasks so that the findings are not confounded by differences across these tasks (e.g., reward type; reinforcement schedule).

A final task design element that is relevant to choice impulsivity concerns the number of choices available to participants. Patros et al. (2017) compared a two-choice (i.e., 1 point after 2 seconds or 20 points after 30 seconds) and five-choice (i.e., 1 point after 10 seconds through 20 points after 50 seconds) task in 8–12-year-old boys with and without ADHD and found that these groups differed in their performance on the two-choice task (*d* = 1.00), but not the five-choice task (*d* = .24). This may indicate that youth with ADHD may not exhibit a more impulsive response style than their TD peers in more complex real-world scenarios with multiple choices. While most studies use a two-choice paradigm, choice impulsivity may depend on contextual variations such as the number of choices available to participants; interestingly, greater choices may better represent hot EF conceptually (i.e., EFs under more ecologically valid scenarios), despite the fact that effect size magnitudes were attenuated.

There are also several gambling task design elements worthy of note. Recent meta-analytic evidence from gambling tasks in youth and adult ADHD suggests that task explicitness does not affect results (Dekkers et al., 2016; Roberts et al., 2021). Specifically, in the meta-regression by Dekkers et al. (2016) and in the meta-analysis of by Roberts et al. (2021), explicitness was not a significant moderator.^2^ The similarity of explicit and implicit gambling tasks is also supported by a systematic review of risk-taking in ADHD that found that 50% of studies using implicit tasks (5/10 studies) and 50% of studies using explicit tasks (2/4 studies) found significant differences between ADHD and TD youth, suggesting that neither task type is superior in its ability to detect effects (Groen et al., 2013). In other words, even when participants are informed of how likely various outcomes are, individuals with ADHD still make suboptimal choices. Relatedly, the extent to which the riskier choice is optimal (i.e., more gains are made) or suboptimal in a given task has also been explored. Recent work found that ADHD individuals differed from controls only if the risky option was also a suboptimal one (Dekkers et al., 2020, 2021), which was replicated in a recent meta-analysis (Roberts et al., 2021). This may suggest the utilization of less complex DM strategies in ADHD, and may be particularly important for understanding the nature of the hot EF deficit; in this case, failure to utilize several (cool) EFs necessary to DM (Dekkers et al., 2020), such as inhibition and working memory.

Lastly, a potentially important task design consideration for all DM tasks is the sufficiency of the “hot” element. Presumably, the possibility of obtaining a reward is what evokes motivation (Homer et al., 2019). While these tasks are considered hot EF due to orbitofrontal cortex involvement in neuroimaging studies (Zelazo & Carlson, 2012), they have not been validated as eliciting emotion or motivation based on other indices, such as self-report, to the authors’ knowledge. Further, DM tasks do not naturally contain a “cool” analog (e.g., DM without reward) to compare with traditional DM tasks (i.e., with reward) in order to estimate the impact of the hot element. Future work would benefit from manipulating the “heat” of DM tasks (Welsh & Peterson, 2014) and considering the success of that manipulation in eliciting motivation.

Collectively, findings indicate that for choice impulsivity tasks, several task design elements are likely to influence findings, including the use of real rewards (particularly when comparing the same choice impulsivity task type; Marx et al., 2018), and that youth with ADHD may behave less impulsively when presented with more than two options (Patros et al., 2017). Further, some task design elements have not been explored (e.g., real versus hypothetical delays) or warrant further investigation (e.g., magnitude of real reward). For gambling, available evidence does *not* suggest that task explicitness influences effect size magnitudes; however, future work parsing the extent to which a decision is risky and/or suboptimal is necessary (Dekkers et al., 2020, 2021; Roberts et al., 2021). Thus, most available DM tasks yield similar effect sizes, but the *mechanisms* of these effect sizes – or the specific task design element that best explains why youth with ADHD perform worse on these tasks – may differ among DM subdomains or task types, warranting further exploration.

#### Decision making: age.

Patros et al. (2016) found that between-group effect sizes (i.e., comparing youth with and without ADHD) for choice impulsivity were similar for children and adolescents (*g* = .45 and .46, respectively).^3^ Similarly, age has not been found to moderate between-group effect size differences on gambling tasks (Dekkers et al., 2016; Roberts et al., 2021). While developmental research in TD populations indicates that DM abilities generally strengthen across development approaching adulthood (e.g., Christakou et al., 2011; Defoe et al., 2015) these data do *not* suggest that age plays a role in youth with ADHD’s DM task performance. In other words, youth with ADHD are impaired in DM compared to TD youth, but these findings suggest that this impairment is similar in magnitude across childhood and adolescence. This is somewhat surprising given theory that ADHD youth demonstrate DM deficits differently across development such that they may be especially risky/impulsive decision makers in adolescence (e.g., Crone et al., 2016; Dekkers et al., 2022; Roberts et al., 2021).

#### Decision making: comorbidity with DBPs.

Several studies have investigated the impact of comorbid DBPs on gambling tasks. Specifically, there is evidence from meta-analyses and reviews that the presence of DBPs exacerbates the magnitude of effect sizes, such that those with ADHD and DBPs performed worse on gambling tasks compared to individuals with ADHD only (*β*_1_= .42, *p* = .07, Dekkers et al., 2016; Groen et al., 2013). Conversely, the role of DBPs in choice impulsivity has not been as consistently studied, with recent meta-analytic reviews (Marx et al., 2018; Patros et al., 2016) failing to examine the potential moderating role of DBPs. However, one review (Pauli-Pott & Becker, 2015) did so, and found that the proportion of ODD/CD cases across studies was *not* associated with effect size differences across both DG and DD tasks. Collectively, these results indicate that the presence of DBPs likely do *not* affect performance on tasks assessing choice impulsivity among youth with ADHD, whereas the presence of DBPs may affect gambling performance among youth with ADHD. Larger magnitude effect sizes on gambling tasks in samples with ODD/CD symptoms is consistent with the idea that DBPs account, at least partially, for a hot EF deficit seen in ADHD (e.g., Dolan & Lennox, 2013; Noordermeer et al., 2020). It also lends credence to the notion that the motivational pathway of the dual pathway model reflects the presence of comorbid DBPs (Pauli-Pott et al., 2019). This pattern of evidence also suggests that gambling tasks are likely assessing a process unique to those involved in performance on choice impulsivity tasks and may be unique to DBPs (e.g., evaluation of *risk*; Matthys et al., 2013).

### Traditionally cool EF tasks adapted to include a hot element (“adapted tasks”)

#### Adapted tasks: task design.

A number of investigators have incorporated̂ emotional or motivational manipulations into (cool) EF tasks to evaluate the impact of these factors on performance. Somewhat surprisingly, tasks integrating an emotional component have largely failed to demonstrate an impact of emotion on overall EF performance in youth with ADHD. For example, among three identified studies utilizing emotional working memory tasks (i.e., two-back tasks integrating positive, neutral, and negative stimuli, such as the International Affective Picture System [IAPS]: Lang et al., 2008; or emotional faces: Passarotti et al., 2010a; Van Cauwenberge et al., 2015; Villemonteix et al., 2017), only one study (Villemonteix et al., 2017) found that youth with ADHD performed worse on the working memory task under emotional conditions. Mixed findings have emerged in studies of emotional STROOP tasks (using IAPS stimuli: Hwang et al., 2015; or emotional words: Passarotti et al., 2010b; Posner et al., 2011; Zhu et al., 2021). Only one study (Posner et al., 2011) has found that youth with ADHD differed from controls in their error rates in an emotional condition (namely, unmedicated ADHD youth demonstrated greater error rates for negative and cognitive conditions as well as greater reaction time in all conditions).^4^ While tasks assessing the impact of emotionally salient conditions on working memory and interference control have largely failed to identify a difference in youth with ADHD, emotional go/no-go tasks using emotional faces or IAPS stimuli have, for the most part, identified an impact of emotionally valanced stimuli on inhibitory processes.^5^ Specifically, several studies find that there are group (ADHD versus TD) by condition (various emotional conditions) interactions, indicating that youth with ADHD respond differentially on tasks assessing emotional interference relative to TD youth.^6^ For example, Tenenbaum et al. (2019) found that youth with ADHD had significantly fewer commission errors, fewer correct go responses, and more correct no-go responses than TD youth during fear conditions compared to control conditions (*d* = .35 to .41), suggesting that ADHD youth struggled to perform optimally during emotionally salient conditions, perhaps due to the greater cognitive resources required, avoidance of fear stimuli, or less overall engagement with emotional stimuli. Another study found that youth with ADHD exhibited impairments in response inhibition (most notably to anger cues) relative to TD youth; in contrast, however, this study found that youth with ADHD demonstrated *greater commissions* for emotional (anger, happiness, sadness) compared to neutral stimuli, most so for anger cues (Köchel et al., 2014). Additionally, ADHD youth had longer reaction times for emotional relative to neutral stimuli, with the longest reaction times exhibited to anger cues and the shortest for happiness cues (Köchel et al., 2014), leading to the conclusion that ADHD youth were impaired in their ability to inhibit responses towards anger cues. Lastly, one study (Karalunas et al., 2020) used drift diffusion modeling and found that for go responses to positive stimuli, adolescents with ADHD had a faster processing efficiency (i.e., greater increase in drift rate; OR = 11.94) and more cautious responding (i.e., increased boundary height; OR = 24.88). For no-go responses to positive stimuli, TD youth increased their drift rate and did not change their boundaries, whereas ADHD youth did not change their drift rate (i.e., slower processing efficiency; OR = 6.09) and reduced their boundary height (i.e., less cautious responding; OR = 24.93; Karalunas et al., 2020). The authors concluded that youth with ADHD experienced increased arousal to positive stimuli, and responded more cautiously to go trials and less cautiously to no-go trials (Karalunas et al., 2020).

Generally, this pattern of findings suggests that emotionally valanced stimuli do not appear to affect working memory or interference control, but do appear to affect inhibition. While specific results differed, all three of these go/no-go studies (Köchel et al., 2014; Karalunas et al., 2020; Tenenbaum et al., 2019) showed that emotion differentially affected youth with ADHD. It could be that emotion affects inhibition in a unique way, or that there are methodological confounds across studies assessing different EF domains. One potential reason for the nonsignificant findings on many of the emotional ATs is that they are limited in statistical power, perhaps due in part to the cool EF task being adapted. For example, the specific working memory tasks utilized in these studies may not be sensitive enough at detecting working memory deficits in ADHD compared to TD youth (i.e., elicit great enough working memory demand; Jusko et al., 2021; Snyder et al., 2015) such that the influence of emotional content on working memory would not be detectable. Similarly, recent evidence suggests that youth with ADHD do not differ significantly from TD youth on the STROOP (Schwartz & Verhaeghen, 2008), though findings are inconsistent (Homack & Riccio, 2004), potentially reflecting differences in the scoring method utilized (Lansbergen et al., 2007). Thus, these cognitive tasks may lack sensitivity due to measurement error (e.g., measurement of multiple cognitive processes in a single task; floor and ceiling effects), resulting in diminished effect sizes (Silverstein, 2008; Snyder et al., 2015). This decrease in effect size (often coupled with small sample sizes; see Supplemental Table 1) reduces power and may cause type two error (Sullivan & Feinn, 2012). Conversely, moderate magnitude between-group differences on go/no-go tasks are fairly consistently detected (Wright et al., 2014). Another potential consideration for emotional ATs is the ability of the “hot” adaptation to evoke emotions. The use of emotional words, such as those used in some emotional STROOP studies, have been criticized as resulting in inconsistent findings and having substantial methodological flaws (Crossfield & Damian, 2021). On the other hand, many of these studies use pictures from the IAPS, which is abundantly used and has been validated cross-culturally (Balsamo et al., 2020; Mikels et al., 2005).

In addition to tasks designed to consider the influence of emotion on EF performance, a number of EF tasks have been adapted to examine the impact of motivation (i.e., rewarding task performance). A review by Ma et al. (2016) examined how reinforcement impacts inhibition and found little evidence that reinforcement differentially affected youth with ADHD (significant interactions between reinforcement condition and group were found in only 24% of studies).^7^ A meta-analysis by Burton et al. (2021) examined this across various clinical populations and TD individuals and found similar results (i.e., reward improved inhibitory control for all participants, but there was no evidence of moderation for any diagnostic group). They also did not find a difference between hypothetical and real rewards (Burton et al., 2021). While most studies included in Ma et al’ (2016) and Burton et al.’ (2021) reviews examined the role of monetary rewards or points (i.e., all but one study included in Burton et al., 2021), some studies specifically examined the role of social reward. For example, Geurts et al. (2008) investigated the effects of social reward on response inhibition (participants were told that they were competing with a peer). While all participants’ performance improved under the rewarding condition and groups’ reaction times were not differentially affected by motivation, youth with ADHD did improve significantly in their accuracy (Geurts et al., 2008). Two studies (Demurie et al., 2016; Kohls et al., 2009) compared the effects of monetary and social rewards. One of these studies (Kohls et al., 2009) found that youth with ADHD showed a greater responsiveness to social reward on a rewarded go/no-go task relative to TD peers (*η*_p_^2^ = .17), whereas the other (Demurie et al., 2016) found that both social and monetary rewards (though particularly, the latter) improved performance for all participants with no differences among ADHD and TD youth on monetary and social incentive delay go/no-go tasks. Additionally, several studies have investigated the role of reward on sustained attention using the CPT (Bubnik et al., 2015; Fosco et al., 2015; Hobson et al., 2011; Rubia et al., 2009a, 2009b). These studies have mostly not detected group differences (Bubnik et al., 2015 detected group differences; Hobson et al., 2011; Rubia et al., 2009a; and Rubia et al.., 2009b did not). Lastly, one study (Fosco et al., 2015) utilized a composite cognition score comprised of inhibition, working memory, and sustained attention and found that reward did improve performance to a greater extent for ADHD participants (*η*^2^= .30), which suggests that perhaps reward influences performance across other EFs (aside from inhibition).

Collectively, while the available evidence suggests that reward influences EF performance, there is little consistent evidence that reward influences performance *more* among ADHD youth. However, this certainly warrants further investigation given evidence that reward may influence EFs more for youth with ADHD depending on EF domain (e.g., Fosco et al., 2015) or reward type (e.g., Kohls et al., 2009). Specifically, it is possible that social rewards are more incentivizing (Wang et al., 2017) or that the salience of a monetary reward may play a role, similar to the findings reviewed above related to DM tasks (e.g., hypothetical versus real reward; reward magnitude).

#### Adapted tasks: age.

The only study to examine the potential moderating influence of age on rewarded AT performance failed to find a significant effect for youth and adults with various psychopathology (Burton et al., 2021). Unfortunately, this has not been examined in youth with ADHD specifically; the review by Ma et al. (2016) examining the impact of reward on inhibition in youth ADHD did not consider the role of age. Further, no studies of emotional or rewarded ATs in ADHD youth have compared performance between children and adolescents to date. Specifically, all but one utilized samples comprised of both children and adolescents and did not specify how results differ; one study (Posner et al., 2011) examined only adolescents. Thus, conclusions regarding the potential moderating influence of age in ATs are not able to be made at this time.

#### Adapted tasks: comorbidity with DBPs.

The reviews by Burton et al. (2021) and Ma et al. (2016) did not examine the role of DBPs. The limited available evidence generally indicates that the presence of ODD/CD does *not* influence results on rewarded ATs, including the stop task (Scheres et al., 2001)^8^ and the CPT (Hobson et al., 2011; Rubia et al., 2009b). On the other hand, DBPs do appear to impact performance on emotional ATs in some studies, though the exact mechanism of influence remains unclear. For example, Zhu et al. (2021) concluded that the presence of DBPs contributed to an *increased* emotional interference effect on the STROOP using emotional words (i.e., slower RTs to negative incongruent stimuli), while Villemonteix et al. (2017) found that ODD was related to *decreased* emotional interference on an emotional working memory task using the IAPS (i.e., ADHD youth had longer reaction times to aversive stimuli than youth with ADHD and ODD). Conversely, however, covarying for ODD and CD did not alter findings in other studies using an emotional go/no-go task (Tenenbaum et al., 2019) and an emotional STROOP task (Posner et al., 2011). Future work is necessary to better understand the role of DBPs on performance on emotional ATs as discrepancies across these studies likely reflect a variety of methodological differences.

## Conceptual and methodological considerations

### Conclusions regarding the role of hot EF in youth ADHD

In order to provide some sense of the hot EF deficit in youth ADHD as a foundational basis for future research, the conclusions that can be made about subdomains and specific task types will be summarized here. Regarding DM, choice impulsivity task types (DD versus DG) reveal a fairly consistent pattern of moderate magnitude between-group differences with youth with ADHD making more impulsive choices than TD youth (Marx et al., 2018; Patros et al., 2016; Pauli-Pott & Becker, 2015). Similarly, gambling task types (implicit versus explicit) result in small-to-moderate effect sizes (Dekkers et al., 2016; Roberts et al., 2021). Additionally, there is a robust literature regarding various design elements that influence the magnitude of effect size differences on DM tasks. Most notably, the number of choices available in choice impulsivity tasks (Patros et al., 2017) and the evaluation of risk and utilization of multiple cool EFs in gambling tasks (Dekkers et al., 2021) are particularly important in explaining the DM deficit in ADHD youth. Additionally, the use of real rewards on DG tasks may reduce choice impulsivity in youth with ADHD (Marx et al., 2018). Further, research suggests that the presence of DBPs may exacerbate risk-taking on gambling tasks (Dekkers et al., 2016; Groen et al., 2013).

While the literature on ATs is much smaller, preliminary patterns emerged. With respect to emotional ATs, ADHD youth demonstrate greater difficulty with emotional inhibition than TD youth, often when negative cues (i.e., fear, anger) are used (e.g., Köchel et al., 2014; Tenenbaum et al., 2019). Notably, ADHD youth do not appear to be differentially impacted by emotional manipulations on other adapted EF tasks (e.g., working memory, interference control), though conclusions are limited due to several methodological limitations (described above). Further, the presence of DBPs appears to influence the magnitude of effect sizes on emotional ATs; however, the direction of influence remains unclear. Finally, while integration of reward improves EF performance, a differential impact for youth with ADHD has not been consistently documented.

Collectively, these findings suggest that ADHD youth show the largest magnitude deficits compared to TD youth for choice impulsivity, emotional inhibition, and gambling (particularly involving the evaluation of risk and especially in the presence of DBPs), and these deficits are stable across childhood and adolescence. However, numerous differences between domains and subdomains considerably limit conclusions regarding the broader hot EF construct.

### Inconsistency in measurement of hot EF

While relatively consistent findings emerge between task types (i.e., comparing DD and DG; comparing implicit and explicit gambling), valuable experimental work has isolated specific components of these tasks that contribute greatly to ADHD-related deficits, and thus may accentuate differences between these tasks in future work. However, there are notable differences *between subdomains* of hot EF. Specifically, choice impulsivity tasks tend to show larger magnitude effect sizes between ADHD and TD youth than gambling tasks (Dekkers et al., 2016; Patros et al., 2016). Further, while increased symptoms of DBPs do *not* appear to impact findings on choice impulsivity tasks (Pauli-Pott and Becker, 2015), they *do* appear to increase the magnitude of effect sizes on gambling tasks (Dekkers et al., 2016; Groen et al., 2013). This suggests gambling tasks invoke processes impaired in youth with DBPs, such as evaluation of *risk* (Matthys et al., 2013), in contrast to the desire for immediate reward assessed by choice impulsivity tasks. This raises concern about the interchangeable use of these tasks in assessing both DM (e.g., Schulze et al., 2021) and the broader hot EF construct. When comparing subdomains of ATs (i.e., emotional versus rewarded ATs), ADHD youth demonstrated impairment on emotional inhibition, whereas there is generally no group differences for rewarded ATs. Further, the presence of comorbid DBPs do *not* appear to moderate the magnitude of effect sizes for rewarded ATs (Hobson et al., 2011; Rubia et al., 2009b; Scheres et al., 2001), but *do* for some emotional ATs (Villemonteix et al., 2017; Zhu et al., 2021), suggesting some differentiation. Additionally, the subdomains of ATs differ in their “heated” stimuli (i.e., emotional words or faces versus the use of reward), unlike the subdomains of DM, which both use reward. Moreover, faces and IAPS stimuli may elicit a different emotional experience than emotional words.

There are also numerous differences *between domains* of hot EF (i.e., DM versus ATs). Most pertinently, the magnitude of between-group differences was generally smaller (and in many cases, not present) for ATs compared to DM tasks. Further, the numerous cognitive processes recruited during DM tasks (Frost & McNaughton, 2017; Murray et al., 2017) are likely different from the discrete domains of EF recruited for a given AT (e.g., working memory). Additionally, DM tasks are only reward-based, whereas ATs can vary depending on the specific heated adaptation (i.e., emotion, reward). Despite the fact that all of these tasks are presumed to assess “hot EF,” the relationships among DM and ATs has not been evaluated directly in youth with ADHD.

Despite the discrepancies described above between domains, between and across subdomains, and between and across task types, the term “hot EF” continues to be used as an umbrella term to characterize functioning in this area in youth with ADHD with limited regard for further nuance. It is possible that they all contribute in some way to a broader hot EF construct, much like working memory, inhibition, and shifting all contribute to (cool) EF (Snyder et al., 2015), but are understood to be unique. No such theoretical model for hot EF exists to the authors’ knowledge. Notably, Figure 1 is not intended to be a proposed model of hot EF; rather, it is intended to illustrate the various distinctions that could be made across commonly used hot EF tasks and to structure the present review. Future work should administer multiple hot EF tasks to the same (adequately large) sample in order to estimate the shared and unique variance across these tasks. While the cool EF construct admittedly benefits from a much longer history and larger research base (e.g., Miyake et al., 2000; Snyder et al., 2015), interest in “hot EF” continues to grow and future research will benefit from a similar approach to that taken by the cool EF literature. Perhaps the largest takeaway from the current review is that a pressing limitation in hot EF research is the use of many disparate tasks under the same label without regard for differences in cognitive demand, emotional experience, and sample characteristics.

While the methodological variability in hot EF research is pressing, it is worth acknowledging that this is not an unprecedented problem in the broader literature interested in cognitive or neuropsychological functioning. For example, task-based versus questionnaire-based assessment of EF (e.g., Smith et al., 2020; Toplak et al., 2013), use of standardized neuropsychological assessments versus experimental cognitive tasks (e.g., Jusko et al., 2021), and variability in task characteristics/complexity (e.g., Molitor & Langberg, 2017; Patros et al., 2019) have all been found to substantially affect findings and preclude firm conclusions about the true magnitude of (cool) EF deficits in youth ADHD. Indeed, some of the measurement issues present in the hot EF literature parallel these broader issues. However, hot EF also demonstrates some unique challenges, particularly the definitional and conceptual ambiguity in the literature, which will be discussed in the following sections.

### Construct validity: emotion, motivation, and their effects on cognition

Given the methodological inconsistency present among hot EF studies, it is important to consider more conceptually what makes a task “hot,” what effect the introduction of motivation or emotion is hypothesized to have on EF, and how findings are expected to inform our understanding of the construct. A review by Chiew and Braver (2011) discussed that while both emotion and motivation have been found to influence EF, research has largely considered these processes separately and has not considered their differences and similarities with respect to their unique and combined effects on cognition. Despite the fact that the most common definition of hot EF is “emotional or motivational salience,” little is known regarding the unique role of emotion on EF. Specifically, all DM tasks and rewarded ATs manipulate only reward or motivation. Unfortunately, the evidence we reviewed does not offer firm conclusions regarding the specific influence of emotion versus motivation given the methodological limitations and scarcity of work on emotional ATs, discussed above. Additional work considering the role of emotion in cognition is critically needed; ATs may be especially helpful in isolating the influence of a specific emotional stimulus on a specific EF domain.

While the tasks discussed herein involve purposeful manipulations of emotion or motivation, it is important to consider the fact that a number of EF tasks are likely to evoke an emotional or motivational experience, even unintentionally. For instance, some have discussed that young children appear to have an emotional experience on game-like conflict tasks such as Head-Toes-Knees-Shoulders (Welsh & Peterson, 2014; Willoughby et al., 2011). While these conflict-type tasks have usually been used as “cool” measures, they may better represent the role of frustration on task performance. In fact, some research highlights the “hot” nature of frustration elicited by conflict tasks and suggests that both hot and cool processes are involved in the resolution of conflict (Willoughby et al., 2011), with some even suggesting a distinction be drawn between hot and cool conflict tasks (Allan & Lonigan, 2011). Furthermore, many studies that do not intend to measure hot EF nor do they utilize conflict-type tasks – for example, working memory or inhibition tasks, which are usually challenging and thus may inadvertently be frustrating – could be tapping into hot EF. Similarly, studies of cool EF that utilize rewards or compensation for participation (e.g., toys, points) may inadvertently invoke motivation. Future research on these unintentional impacts on EF performance is warranted.

In order to isolate and understand the influence of emotion and motivation, careful thought should be applied to the optimal measurement of these processes as well as how to identify their effect on EF. A critical feature lacking from much of the reviewed hot EF research is exploration of the role of emotional or motivational context in comparison to a “cool” context in order to derive greater insight regarding their influence. This would allow comparison of hot and cool conditions while holding cognitive demands constant in a single sample with given characteristics (age, personality) that may impact the experience of “heat” (Welsh & Peterson, 2014). Relatedly, future studies will benefit from verifying the extent to which their sample perceives the context as being emotionally or motivationally salient (e.g., as was done by Van Cauwenberge et al., 2015). Finally, clarity with respect to what it *means* for a heated component (e.g., emotion, motivation) to impact performance is warranted. For example, it is generally expected that youth with ADHD will make riskier or less advantageous decisions when completing DM tasks, whereas reward is often thought of as something that will improve performance on a rewarded AT. Similarly, for emotion, the adaptation could presumably improve or worsen performance, depending on the valance (positive or negative) of the emotional stimuli. Clarity regarding expected outcomes *a priori* would strengthen the literature and provide a better understanding of the mechanisms underlying hot EF performance.

### Hot EF’s distinguishability from related constructs

“Hot EF” encompasses a wide array of tasks, which are often assumed to be “hot” by design despite little evidence that emotion or motivation was sufficiently evoked. Often, a task designed to assess for a given construct is assumed to do so successfully without firm empirical grounding. For instance, some “working memory” tasks are assumed to successfully elicit working memory by design, despite evidence that some tasks are more sensitive than others (Conway et al., 2005; Jusko et al., 2021; Wells et al., 2018). This conflation of *task* with *construct* (Nigg, 2017) is a widespread problem and has implications for the hot EF literature. Furthermore, hot EF interfaces with other constructs such as self-regulation (SR), effortful control (EC), cognitive control (CC), and emotion regulation (ER), which all have been criticized on similar grounds (Nigg, 2017; Peterson & Welsh, 2014; Zhou et al., 2012). While this literature is beyond the scope of the current review, there are a few salient points to be made with regard to hot EF.

In a review of these terms, Nigg (2017) discusses that the hot EF construct arose from interest in the top-down regulation of emotion, in which case it is unclear whether hot EF differs from self-regulation (i.e., the way that cool EFs are recruited to assist in SR). While this may be the original intention of hot EF theoretically, it is unclear if this is the consensus among studies of hot EF (i.e., that hot EF is, or *only* is, the regulation of affective information in pursuit of goal-directed behavior). As illustrated by the above discussion, the hot EF tasks reviewed herein are likely not all identifying the same construct. Thus, depending on the specific task used, hot EF seems to encompass different processes (and also, corresponding to which process, overlaps more or less with various other related terms).^9^ For example, for DM tasks, choice impulsivity reflects the preference for immediate rewards, focusing on the *temporal* features of a choice.^9^ This aspect of impulsivity is related to self-control, or the ability to choose larger delayed rewards over smaller immediate ones.^9^ This is in contrast to gambling tasks, which focus on the probability features of a choice.^9^ Emotional ATs, if conceptualized as the ability to suppress task-irrelevant information in order to perform on an EF task, may be thought of as involving interference control, a type of inhibition.^9^ Further, this relates to cognitive control, which modulates conflict (with primed responses, task-irrelevant information, or goal conflict such as task-switching).^9^ On the other hand, if emotional ATs are conceptualized as tasks which elicit emotions that have to be regulated in favor of goal-directed action, it may be more akin to ER.^9^ Lastly, rewarded ATs do not require this kind of suppression of irrelevant information to perform well; instead, they merely incentivize participants to perform optimally.

Although the exact way that hot EF is similar or different from related constructs depends upon the specific hot EF task utilized, one consistency among hot EF tasks that can be identified is that they recruit EFs. Just as EFs can be used to facilitate SR, but are not *only* SR (Nigg, 2017), these related constructs (SR, ER, CC, EC) do not always recruit EFs or focus primarily on EF the way that a hot EF task does. Hot EF is not unique in its conceptual ambiguity, and resolving each construct’s unique contribution to psychological science is a multidisciplinary effort. Further isolation and identification of the components of hot EF, much like Nigg (2017) provided for SR, is critically needed.

### Hot EF’s distinguishability from cool EF

Questions persist regarding the extent to which hot and cool EF differ from one another. The majority of studies that have considered this question are rooted in the SR literature (i.e., preschoolaged nonclinical youth; Garon, 2016; Welsh & Peterson, 2014); however, their findings are important to discuss as they are the only evidence that provides some insight into this question. Some studies found no distinction between hot and cool EF in correlations and factor analyses (e.g., Carlson & Wang, 2007; Prencipe et al., 2011; Sulik et al., 2010; Thorell, 2007); others found divergent patterns of correlations or two factors (e.g., Brock et al., 2009; Kim et al., 2013; Mulder et al., 2014); and some found that one- and two-factor models show an equally good fit (Allan & Lonigan, 2014; Denham et al., 2012). These discrepancies may reflect differences in task selection (Welsh & Peterson, 2014). For example, the Iowa Gambling Task (IGT) tends to correlate more with cool EF tasks, whereas delay tasks exhibit the opposite pattern; unsurprisingly, this results in findings that indicate that delay tasks and the IGT tend not to correlate with one another (Garon, 2016; Prencipe et al., 2011). Additionally, many of the studies supporting a unitary conceptualization relied on “conflict” inhibition-type tasks to assess cool EF (e.g., Simon Says, STROOP, knock-tap; Carlson & Wang, 2007; Sulik et al., 2010; Thorell, 2007). Given that conflict tasks may unintentionally elicit frustration (Willoughby et al., 2011) and, to that end, may involve some “hot” processes (Allan & Longinan, 2011; Welsh & Peterson, 2014), conflict tasks might not be sufficiently different from other measures presumed to assess “hot EF.” Collectively, these differences highlight the need to further evaluate the extent to which gambling tasks may be more strongly associated with cool EF, whereas conflict-type “cool EF” tasks may be more strongly associated with hot EF in youth ADHD. This work would improve our understanding of the discrepant literature regarding hot and cool EF’s separability.

At a more conceptual level, it is unclear to what extent hot and cool EF *should differ.* Many have suggested that “hot” and “cool” EF are not distinct processes but rather, “similar cognitive functions that fall along a continuum of emotional activation” (Homer et al., 2019), and that in most situations, hot and cool EF are both involved (Garon, 2016; Homer et al., 2019; Perone et al., 2018; Zelazo, 2020). Hot EF – containing both emotional or motivational elements as well as some EF component – may show similar magnitude correlations with both strictly affective and strictly cognitive variables. As a matter of fact, the present review highlights that hot EF effect sizes in ADHD are at least partially dependent upon cool EF processes. For example, youth with ADHD may demonstrate greater choice impulsivity in the face of hypothetical rewards (potentially reflecting deficits in advanced planning) and make more suboptimal choices on gambling tasks (potentially reflecting deficits in cool EFs required for optimal decision making). While the present review focuses only on behavioral task performance, incorporating multiple levels of analysis may be helpful with several of the issues delineated here, such as determining the effects of task design elements, the extent to which emotion and motivation is elicited, and hot EF’s relationship with related constructs.

### Level of analysis

As briefly noted above, self-report will likely be beneficial for gauging the extent to which participants perceive a task as emotionally or motivationally salient. Additionally, this approach allows for the acquisition of data on participants’ baseline self-reported emotional lability, response to motivation and sensitivity to reward, and perceived cognitive abilities, as participants likely differ along across these areas which may impact performance on a given task. Physiological data are likely also to advance the literature on hot EF. For example, studies have found that youth with ADHD (and DBPs) show greater difficulty on emotional go/no-go tasks compared to TD youth that correspond with irregularities in para-sympathetic-based emotion regulation (Tenenbaum et al., 2019). Physiological data could further inform behavioral data and clarify how tasks elicit similar or different experiences and responses. For example, if two tasks demonstrate similar effect sizes for behavioral data, but one measure elicits a unique physiological response, this highlights the tasks’ distinguishability (Campbell & Fiske, 1959). Lastly, neuroimaging has great potential to advance research in this area. Extant work examining the neurobiology of hot EF in ADHD and related externalizing disorders has shown that hot and cool EF are distinct such that cool EF is associated typically with the dorsolateral prefrontal cortex, while hot EF is typically associated with the orbitofrontal cortex and its connections with the limbic system (Castellanos et al., 2006; Skogli et al., 2017; Tsermentseli & Poland, 2016). Additional neuroimaging research will help clarify the interface of “top-down” and “bottom-up” processes (Cromwell & Panksepp, 2011; Sarter et al., 2001) and their measurement as it relates to disentangling EF, hot EF, SR, EC, and ER (Nigg, 2017). Given recent acknowledgement that many theoretical terms – such as cognition, emotion, and motivation – were formed before recent developments in neuroscience, the field will benefit from transitioning from being strictly theoretical to basing these constructs in a neurobiological reality (Cromwell & Panksepp, 2011).

### Limitations of present review

The present review synthesizes the literature on hot EF in youth with ADHD, highlights gaps in the literature, and charts future directions for addressing methodological and conceptual challenges. Importantly, the present review was not a systematic review or meta-analysis. Instead, we conducted a critical review in which we attempted to provide a detailed and objective overview of the evidence base followed by a theoretical discussion that evaluates the quality of this work, describes key takeaways, and outlines limitations for future research to address. However, this approach did not allow us to provide quantitative evidence, and it is certainly possible that studies were missed. It is our hope that this review provides a foundation for future work on hot EF in youth ADHD and may provide precedent for future meta-analyses. Additionally, the present review did not examine the role of different ADHD presentations given the scarcity of this consideration in the literature reviewed (i.e., only 3 of 10 meta-analyses/reviews and 4 of 24 individual studies investigated the role of ADHD presentation).

### Future directions

The most pressing issue in the literature on hot EF is the use of such widely varied tasks to assess for the construct. Additional work should evaluate the extent to which various tasks correlate with one another and are reflected by a latent hot EF construct. Relatedly, future work should consider the extent to which hot and cool EF differ in youth with ADHD, keeping in mind that they may not be opposites but rather, work together (Perone et al., 2018). Additionally, the hot EF literature would benefit from directly comparing the influence of emotion and motivation in the same sample, and also comparing participants’ performance on the EF task before and after incorporation of experimental manipulations (Chiew & Braver, 2011). Along these same lines, researchers should specify what outcomes are expected after successful induction of “heat” (i.e., improved or impaired performance) and what that means for our understanding of hot EF in youth with ADHD. Further, future work should consider the role of subjective experience (Chiew & Braver, 2011), both for studies with an explicit goal of assessing hot EF, and for studies that may do so inadvertently (e.g., cool EF studies utilizing incentives for overall performance). Pre- and post-ratings of mood, frustration, and motivation will likely facilitate this goal. In addition to self-report, incorporation of additional levels of analysis (e.g., physiology, neurobiology) is likely to further our understanding of the extent to which various tasks elicit similar or different responses in individuals independent of their task performance. Additionally, future work on hot EF would benefit from examining the role of ADHD presentations given literature suggesting that hyperactivity/impulsivity may have a stronger relationship with hot EF and DBPs (e.g., Pauli-Pott et al., 2019). Relatedly, future work is warranted that explores if and how DBPs influence effect sizes on emotional ATs but not rewarded ATs. Finally, research on the impact of age on ATs as well as what type of reward may elicit age-related differences in performance (e.g., adolescents may be more influenced by social rewards; Galvan, 2010) is needed.

Hot EF has the potential to be incredibly useful for better understanding the etiology of ADHD, the comorbidity of ADHD with DBPs, and functional impairments associated with hot EF deficits in youth with ADHD. More broadly, hot EF research is uniquely well-suited to address a pressing limitation in psychological research; namely, a lack of understanding about the influence of emotion and motivation on cognition (Chiew & Braver, 2011). Despite its promise, poor conceptual clarity and inconsistent measurement of hot EF persist. Unfortunately, these limitations make it difficult to draw many meaningful conclusions about hot EF in ADHD after 20 years of research. This conceptual and methodological ambiguity also precludes future advancement despite the term’s growing popularity. We urge that any future research on hot EF prioritizes basic work (i.e., how hot EF should be measured; how various hot EF tasks differ from one another; how hot EF meaningfully differs from other constructs) over applied work (i.e., treating hot EF as an established term to evaluate the author’s hypotheses), as the term “hot EF” offers little utility as it is currently understood and utilized. However, with greater conceptual and methodological consensus, hot EF may prove to be a useful construct for understanding the intersection of cognition, motivation, and emotion as well as etiological pathways to ADHD and related disorders.

## Supplementary Material

Supplementary material

**Supplementary material.** For supplementary material accompanying this paper visit https://doi.org/10.1017/S0954579422001432

## Footnotes

1 Cohen’s *d* and Hedge’s *g* are both SMD statistics. While there are some differences in calculation, rule-of-thumb interpretation of Cohen’s *d*, Hedge’s *g*, and SMD effect sizes are all .20 = small, .50 = medium, and .80 = large (Andrade, 2020; Cohen, 1992; Durlak, 2009; Ellis, 2010).

2 In the Dekkers et al. (2016) meta-regression, implicit tasks *did* demonstrate a significant effect size comparing ADHD and TD youth and explicit tasks did *not*. Authors discuss that nonsignificant between-group effects for explicit tasks may be due to the fact that fewer explicit tasks were available for analysis, limiting power.

3 While the meta-analysis by Patros et al. (2016) determined that age *was* a moderator, this was likely due to the fact that effect sizes were considerably larger for preschoolers (*g* = .83). This is particularly important as existing hot EF reviews have focused on preschoolers (Garon, 2016; Welsh & Peterson, 2014) and may come to very different conclusions regarding the role of age. On the other hand, studies of gambling that compare ADHD and TD youth examine only children and adolescents (e.g., Dekkers et al., 2016, Groen et al., 2013) with most gambling tasks for preschoolers only in nonclinical samples (e.g., see Garon, 2016).

4 Passarotti et al. (2010b) found a group (ADHD, TD, bipolar) by condition (negative, positive, neutral) interaction for reaction time; when comparing ADHD and TD youth, those with ADHD had a slower reaction time in all conditions (i.e., was nonspecific to the hot adaptation). No significant findings for accuracy emerged. Zhu et al. (2021) found that ADHD and ADHD + DBP groups had slower RTs on various conditions compared to TD youth (see Supplemental Table 1 for details); authors did not describe accuracy findings.

5 For an exception, see López-Martín et al., 2015; details provided in Supplemental Table 1.

6 The present review considers only behavioral task data. Many studies utilizing ATs also consider physiology or neurobiology.

7 This study utilized both a qualitative and quantitative approach. In a subset of studies eligible for meta-analysis, they found that reinforcement-induced improvement in inhibition was larger for the ADHD group compared to the control group, such that ADHD youth normalize their performance to the baseline level of controls. For a detailed discussion of differences between qualitative and quantitative reviews, see Ma et al. (2016).

8 DBPs did not influence stop signal reaction time or accuracy. Those with ADHD, ODD/CD, and ADHD + ODD/CD had slower and more variable reaction times compared to TD youth, and ADHD + ODD/CD youth had a slower speed of response execution than TD youth; however, is not clear if these findings are due to the presence of ADHD.

9 Throughout the discussion on hot EF’s distinguishability from related constructs, definitions of impulsivity, inhibition, EF, EC, SR, and ER are from Nigg (2000) and Nigg (2017) reviews.
