# Supplementary material for: A critical review of hot executive functioning in youth attention-deficit/hyperactivity disorder: Methodological limitations, conceptual considerations, and future directions
Source: Dev Psychopathol. Author manuscript; Available in PMC 2025 Sep 9. (PMC12419674; doi:10.1017/S0954579422001432)
Supplement: Supplementary material [file NIHMS2106540-supplement-Supplementary_material.docx]

**Supplemental Table 1*.*** *Summary table of studies discussed in the review of literature*

| **Study** | **Domain (Subdomain)**^a^ | **ADHD sample** | **DBPs** | **Age of sample** | **Tasks utilized** | **Findings** |
| --- | --- | --- | --- | --- | --- | --- |
| **Systematic Reviews & Meta-Analyses Included in Review** | | | | | | |
| Burton et al., 2021 | AT(R)^b^ | *k*=17 out of *k*=80 had ADHD group^c^. | *k*=1 had ODD group. | Included youth and adults; *k*=28 were youth samples. | Go/no-go, Stop Task, Flanker, Simon, STROOP, Anti-saccade. | Reward improves inhibitory control, but with no moderation by group (incl. comparing ADHD and TD), age (youth vs. adults), or reward type (monetary, points, other). |
| Dekkers et al., 2016 | DM(G) | ADHD vs. TD *k*=52: ADHD *n*=1175*;* TD *n*=1222. 16 additional studies incl. to investigate DBD/APD moderation (total *k*=75). | *k*=40 had comorbid DBD/APD, *k*=27 had comorbid ADHD in DBD/APD samples. | *k*=49 of *k*=75 with M_age_<18. | IGT, HDT, FPGT, CT, DOT, RDT, BART, CBG, GDT, CGT, PDT, GMT, JPT, RDMUR. | ADHD more risky than TD on DM(G). Studies with higher % DBPs had (insignificantly) larger effect sizes. No moderation for age (children vs. adolescents vs. adults). Implicit (and not explicit) tasks yielded a significant between-group effect size; explicitness was not a moderator. |
| Dekkers et al., 2021 | DM(G) | ADHD *n*=1144, TD *n*=1108. | *k*=3 of *k*=48 had DBPs in group comparisons (2 DBD, 1 ODD). | *k*=33 of *k*=48 with M_age_ <18. | IGT, FPGT, GDT, GMT, CGT, HDT, JPT, PDT, GT, CP. | ADHD and TD differed if the risky option was suboptimal, and did not differ if the risky option was *not* suboptimal. Meta-regression did not examine role of DBPs or age. |
| Groen et al., 2013 | DM(G) | Systematically reviewed 14 studies of youth ADHD. | Of 14 studies of youth, 3 had DBPs in group comparisons (1 ODD, 1 CD, 1 ODD/CD). | Discusses separately findings for youth and adults; 14 studies of youth are reviewed. | IGT, DOT, BART, CGT, GDT, MMG, PDT. | 10 studies with implicit tasks; 4 studies with explicit tasks. 5/10 implicit studies found significant group effects; 2/4 explicit studies found significant group effects (such that ADHD group made riskier decisions than TD group). DBPs exacerbated group differences. Did not compare children and adolescents. |
| Ma et al., 2016 | AT(R)^b^ | Quantitative review w/o outliers: ADHD *n*=210, TD *n*=274. Quantitative review w/ outliers: ADHD *n*=353, TD *n*=371. | 2 of 17 studies across qualitative and quantitative reviews had DBPs in group comparisons (1 ODD/CD, 1  disruptive). | All studies examined only youth 6-18 years. | Go/no-go, Stop Task. | Significant interactions between reinforcement condition and group found in 24% of all studies. In a subset of meta-analyzed studies (10/17), reinforcement-induced improvement in inhibition was larger for ADHD (performance normalizes to TD levels). Did not investigate role of DBPs. |
| Marx et al., 2018 | DM(CI) | Total *n*=3763, ADHD *n*=1986. | Did not provide information on DBPs in included studies. | SCP studies: 19 youth, 3 youth+adults, 0 adults. TDP studies: 8 youth, 1 youth+adult, 6 adults. | CDT; MIDA; TDP. | Both SCP (i.e., DG) and TDP (i.e., DD) had small-to-medium group effects (ADHD more choice impulsive than TD). Real rewards in DG tasks reduced CI in ADHD group. Did not investigate role of DBPs. Age was not a significant moderator for DG (children vs. adolescents+adults) or DD (children+adolescents vs. adults). |
| Patros et al., 2016 | DM(CI) | ADHD *n*=2360, TD *n*=1960. | Did not provide information on presence of DBPs; stated that 78% of included studies examined samples with comorbidities. | All studies examined only youth 3-17 years. | Categorized all CI findings into DD and DG tasks; did not provide specific task names. | ADHD more choice impulsive than TD (DD and DG). Age moderated effect sizes; preschool effect sizes were much larger, whereas child and adolescent effect sizes were similar and moderate. Did not investigate role of DBPs. |
| Pauli-Pott & Becker, 2015 | DM(CI) | ADHD *n*=1395, TD *n*=1195. | *k*=18 of *k*=23 provided information on ODD/CD comorbidity. | All studies examined only youth 5-18 years. | Naturalistic Choices, Response Delay Bias, CDT, MIDA, DD^d^. | ADHD more choice impulsive than TD. Child effect sizes larger than adolescent effect sizes, though age was not a significant linear (dimensional) moderator. % ODD/CD not associated with effect sizes. |
| Roberts et al., 2021 | DM(G) | ADHD *n*=2577, TD *n*=2606. | *k*=57 of 82 included DBD. | *k*=48 of *k*=82 had M_age_ <18. | IGT, FPGT, GDT, BART, DOT, GMT, CGT, PDT, CT, CP, CDT, HDT, RDMUR, JT, RDT. | ADHD more risky than TD on DM(G). Age, inclusion of ODD/CD, explicitness, number of choices, reward type, and feedback were *not* significant moderators. Effects were larger when the risky choice was suboptimal (vs. advantageous). |
| Schulze et al., 2021 | DM(CI+G) | ADHD-C *n*=193, ADHD-IN *n*=265, ADHD-H/I *n*=231. | 1 study used ODD as a comparison group instead of TD. DBPs otherwise not mentioned. | 7 studies with M_age_<18; 1 study with M_age_=22.3. | IGT, GDT, MMG, TDP, Probabilistic Game Task. | ADHD-C<TD, ADHD-IN<TD, ADHD-H/I<TD on DM (i.e., poorer performance, more impulsive/risky). Age did not moderate results. Did not investigate role of DBPs. |
| **Individual Empirical Studies Included in Review** | | | | | | |
| Bubnik et al., 2015 | AT(R) | ADHD *n*=25, TD *n*=33. | *n*=11 and *n*=2 ADHD youth had ODD and CD, respectively. | 9-12 years. | CPT (+/- points in exchange for prizes). | Reinforcement improved performance (hits) for all; reinforcement improved performance *more* for ADHD youth than TD youth. Reinforcement reduced ADHD false alarm rate; TD youth had few false alarms in either condition. ADHD and TD youth did not differ in vigilance, so role of reinforcement was not investigated. Did not investigate role of DBPs. |
| Dekkers et al., 2020 | DM(G) | ADHD *n*=81, TD *n*=100. | 30% of ADHD youth met criteria for ODD and/or CD. | 12-19 years. | GMT with modifications for risky vs. suboptimal; decision-strategy; feedback. | ADHD and TD youth did not differ when risky and safe options were equally advantageous. Youth with comorbid DBD had riskier DM on loss-probability GMT, not gain-probability GMT. ADHD youth used less optimal DM strategies than TD youth; DBD did not impact DM strategy complexity. ADHD and TD youth improved similarly with feedback (i.e., no group differences), with and without DBD. |
| Demurie et al., 2016 | AT(R) | ADHD *n*=34, ASD *n*=36, ADHD+ASD *n*=16, TD *n*=41^c^. | *n*=16 of ADHD and *n*=6 of ADHD+ASD met ODD criteria; none met CD criteria. | 8-16 years. | Monetary Incentive Delay (MID; go/no-go with low/high monetary reward); Social Incentive Delay (SID; go/no-go with low/high points and pictogram feedback); monetary TDP (hypothetical). | Main effect of reward amount for omission errors and RT of correct go for all participants. Main effect of reward type: fewer omissions and faster go RT in monetary conditions. ADHD and TD did not differ in overall task performance. No group x reward type interactions. Did not investigate the role of DBPs. |
| Fosco et al., 2015 | AT(R) | ADHD *n*=25, TD *n*=33. | 45% ODD and 31% CD in the ADHD sample. | 9-12 years. | Composite of Stop Task; n-back; CPT (each +/- points in exchange for prizes). | Diagnosis x reinforcement interaction indicates a stronger influence of reinforcement on cognitive composite in ADHD group than TD group. |
| Geurts et al., 2008 | AT(R) | ADHD *n*=22, TD *n*=33, ASD *n*=22^c^. | *n*=6 members of the ADHD group had comorbid ODD (no CD). | 8-13 years. | Adapted Flanker (SpongeBob Flanker +/- peer competition). | MRT: ADHD significantly slower than TD, no group x motivation interaction. Accuracy: ADHD less accurate than TD (insignificantly); group x motivation interaction significant. All children improved significantly with reinforcement (speed and accuracy). Did not investigate the role of DBPs. |
| Hobson et al., 2011 | AT(R)^e^ | ADHD±ODD/CD *n*=31, ODD/CD only *n*=28, TD *n*=34. | Included as groups of interest. ADHD+ODD/CD *n*=31, ODD/CD only *n*=28, TD *n*=34. | 10-17 years. | Go/no-go^*^, Stop Task^*^, Switch^*^, WMST^*^, CPT (+/- points), IGT. | There were no significant group x reward interactions with any CPT variables. |
| Hwang et al., 2015 | AT(E) | ADHD *n*=26, TD *n*=35. | 1 ADHD child had comorbid ODD. | ADHD M_age_=14.53 (SD=2.00), TD M_age_=13.91 (SD=2.13). | Affective STROOP (IAPS: negative, neutral, positive). | RT: ADHD slower than TD, incongruent slower than congruent, negative slower than neutral. No interactions. Accuracy: incongruent slower than congruent. No group or emotion effects and no interactions. Removal of children with comorbidities (1 ODD, 2 SUD) had a “minimal impact” on results. |
| Karalunas et al., 2020 | AT(E) | Total *n*=130, ADHD *n*=61. | DBPs not listed as exclusion criteria, but not otherwise mentioned. ADHD group had higher parent and teacher Conners’ Aggression scores than TD group. | ADHD M_age_ =13.9 (SD=1.5), TD M_age_=13.8 (SD=1.1). | Emotional go/no-go (NimStim faces: happy, fear, and neutral). | Go responses to positive stimuli: ADHD faster processing efficiency and more cautious responding than TD. No-go responses to positive stimuli: ADHD youth slower processing efficiency and less cautious responding than TD. Did not investigate role of DBPs. No main effects of emotion or group x emotion interactions for fear condition. |
| Köchel et al., 2014 | AT(E) | ADHD *n*=16, TD *n*=16. | Children with ODD or CD diagnoses were excluded from the study. ADHD group had higher parent-rated SDQ conduct problems than TD group. | 8.5-11.8 years. | Emotional go/no-go (Karolinska Directed Emotional Faces: angry, happy, sad). | Commissions: main effect of emotion (emotional>neutral). Main effect of group (ADHD>TD). Emotion x group interaction: ADHD greater commissions than TD for anger cues. Omissions: main effect of emotion (emotional>neutral). No significant group effect or interactions. RT: main effect of emotion (emotional>neutral). No significant group effect. Emotion x group interaction significant: longer RT for angry compared to sad faces in ADHD group. RTV: main effect of emotion (emotional>neutral). Main effect of group (ADHD>TD). No emotion x group interaction. Did not investigate role of DBPs. |
| Kohls et al., 2009 | AT(R) | ADHD *n*=16, TD *n*=16. | 2 ADHD youth had comorbid ODD, none had comorbid CD. | 8-13 years. | Incentive go/no-go task (+/- monetary or social reinforcement). Monetary: picture of wallet with 50 eurocent or empty wallet; Social: happy face or neutral face. | Significant effect of reward; no significant effect of group; significant group x reward interaction. For false alarm rates, ADHD youth were more responsive to social (and not monetary) rewards than TD youth. For RT, ADHD youth responded slower (and less impulsively) with monetary reinforcement, whereas TD youth responded faster with monetary reinforcement. |
| López-Martín et al., 2015 | AT(E) | ADHD *n*=24, TD *n*=24. | Four youth had comorbid ODD; no other comorbidities present. | 8-13 years. | Emotional go/no-go (IAPS: negative, neutral, and positive). | No effect of group, emotion, or group x emotion interactions. Analyses were conducted without the four ODD youth, and similar results were obtained. |
| Passarotti et al., 2010a | AT(E) | ADHD *n*=14,  Bipolar *n*=23, TD *n*=19^c^. | No mention of DBPs. | 10-18 years (M_age_=13.36, SD=2.55). | Emotional 2-back (Gur faces: happy, angry, and neutral). | RT: main effect of emotion (angry slower than neutral). No group effect or interactions. Accuracy: ADHD less accurate than TD. No effect of emotion or interactions. |
| Passarotti et al., 2010b | AT(E) | ADHD *n*=15,  Bipolar *n*=17, TD *n*=14^c^. | No mention of DBPs. | 10-18 years M_age_=13.78 (SD=2.47). | Emotional STROOP (ANEW words: negative, positive, neutral). | No significant findings for accuracy. ADHD RT negative<positive words. ADHD<TD RT on negative, positive, and neutral words. |
| Patros et al., 2017 | DM(CI) | ADHD *n*=16, TD *n*=23. | 6 ADHD youth had comorbid ODD; no mention of CD. | 8-12 years. | Two-choice impulsivity task and five-choice impulsivity task. | ADHD youth were significantly more impulsive than TD youth on the two-choice, but not five-choice, task. |
| Posner et al., 2011 | AT(E) | ADHD *n*=15, TD *n*=15. | ODD and CD free to vary. | 11-16 years (ADHD M_age_=13.5, SD=1.2; TD M_age_=13.4, SD=1.2). | Cognitive STROOP*, emotional STROOP (positive and negative words). | ADHD youth (unmedicated) had slower RTs than TD youth in all conditions (cognitive, negative, positive) and greater errors than TD youth under negative and cognitive (not positive) conditions. Covaried for the presence of ODD and CD. |
| Rubia et al., 2009a | AT(R) | ADHD *n*=13, TD *n*=13. | ODD and CD free to vary; ODD/CD present in one individual. | 10-16 years. | Rewarded CPT (monetary: one of two target letters was rewarded). | No effect of reward on omission errors for either group; no reward x group interactions. |
| Rubia et al., 2009b | AT(R) | ADHD *n*=18,  CD *n*=14, TD *n*=16. | Included as groups of interest. CD *n*=14; all CD youth met criteria for ODD. ADHD youth were “non-comorbid.” | 9-16 years. | Rewarded CPT (monetary: one of two target letters was rewarded). | Significant effect of reward for all groups on omission errors and RTV (both lower during reward). No significant group effects or group x reward interactions. |
| Scheres et al., 2001 | AT(R) | ADHD *n*=24, ODD/CD *n*=21, ADHD+ODD/CD *n*=27, TD *n*=41. | Included as groups of interest. ODD/CD *n*=21, ADHD+ODD/CD *n*=27. | 7-12 years. | Stop Task (+/- points in exchange for prizes and praise from experimenter). | Main effect of reward for SSRT, MRT, % correct go responses. Reward did not effect RTV. No group differences or group x reward interaction for SSRT. No group differences in accuracy. Significant group effect for MRT and RTV (all clinical groups slower and more variable than TD). Group x reward interaction: ADHD+ODD/CD slower speed of response execution than TD. |
| Scheres et al., 2006 | DM(CI+G) | ADHD *n*=22, TD *n*=24. | 7 ADHD youth met criteria for ODD; CD not mentioned. ADHD youth had higher parent-rated CBCL Aggression and Conners’ Oppositional scores than TD youth. | 6-17 years; compared children (6-11) and adolescents (12-17). | TDP (immediate variable reward of 0-10 cents vs. large constant reward of 10 cents after variable delay of 0-30 seconds); PDT (10 cents at probability of 0, .25, .5, .75, 1, vs. certain reward of 0-10 cents). | ADHD and TD youth did not differ on TDP with or without post-reward delays; all youth made choices to maximize gains. Children discounted delays significantly more than adolescents both with and without post-reward delays. All youth discounted delayed rewards more when doing so shortened task duration. Task version, age, and group did not interact. No significant effect of group, age, or group x age interaction on PDT. Did not explore the role of DBPs. |
| Scheres et al., 2010 | DM(CI) | Total ADHD *n*=45 (ADHD-C *n*=25, ADHD-IN *n*=20), TD *n*=37. | No mention of DBD diagnoses. ADHD-C and ADHD-I groups both had higher parent-reported CBCL ODD and DBD-RS ODD scores than TD group. | 6-17 years. | Three TDP tasks with varied reward magnitude (between 2 and 8 cents now or between 5 and 10 cents later) and session length (40-80 trials). | Main effect of group: steeper discounting in ADHD-C compared to TD and ADHD-IN. No group x task type interaction. |
| Tenenbaum et al., 2019 | AT(E) | ADHD *n*=75, TD *n*=91. | 69.3% ODD and 9.30% CD in ADHD group. | 5-13 years. | Neutral go/no-go*, Emotional Go/no-go (NimStim faces: fearful and calm). | ADHD fewer commission errors, fewer correct go responses, more correct no-go responses than TD youth during fear conditions compared to neutral conditions. Main effect of emotion and group for omissions, but no interaction. Covaried for ODD and CD in all analyses; overall results did not differ with covariates. |
| Van Cauwenberge et al., 2015 | AT(E) | ADHD *n*=39, TD *n*=44. | *n*=11 youth had comorbid ODD, no mention of CD. | 8-15 years. | Emotional n-back (0-back and 1-back; IAPS pictures: negative, neutral, positive, and blank screen). | RT: emotional slower than neutral pictures; 1-back slower than 0-back; ADHD slower than TD. Group x distractor type interaction not significant (nor were any other interactions). Exclusion of ODD youth did not substantially change results. |
| Villemonteix et al., 2017 | AT(E) | ADHD *n*=33, TD *n*=24. | *n*=7 ADHD youth had comorbid ODD, no mention of CD. | 8-13 years. | Emotional n-back (1-back, 2-back; IAPS pictures: negative, neutral, and positive). | Group x emotion interaction: ADHD slower RT than TD in presence of negative, not neutral, distractors. Covariance for ODD increased effect sizes on RT findings (i.e., associated with decreased emotional interference). |
| Zhu et al., 2021 | AT(E) | ADHD *n*=30, DBD *n*=26, ADHD+DBD *n*=22, TD *n*=20^f^. | Included as groups of interest. DBD *n*=26, ADHD+DBD *n*=22. | 9-16 years. | Golden’s STROOP*, Emotional STROOP (positive, negative, and neutral words)^g^. | ADHD longer MRT than TD for positive-congruent. ADHD+DBD and ADHD longer MRT than TD for positive incongruent, negative congruent, and neutral. ADHD+DBD and DBD longer MRT on negative incongruent compared to TD. Authors conclude DBDs contributed to increased emotional interference. Errors for emotional Stroop not described. |

*Note.* Discussion of DM was largely dependent on numerous extant meta-analyses and reviews, except for individual empirical studies that were worthy of closer examination as they directly tested one of the main areas of focus (i.e., task design, age, DBPs). Discussion of the effects of reinforcement on inhibition (one task type of AT[R]) were similarly dependent on by Burton and colleagues (2021) and Ma and colleagues (2016) reviews, except for notable studies. All other discussion of adapted tasks (i.e., other AT[R] tasks, all AT[E] studies) were dependent upon individual empirical studies. Listed studies may have utilized various methods (e.g., physiology, neuroimaging, effects of methylphenidate) and included numerous analyses (e.g., all main effects and interactions); Table 1 only included findings most relevant to the present review for brevity. Presentation of information across rows varies due to reporting differences across studies. Asterisks indicate that a task listed as included in a given study was not a hot EF task.
*Abbreviations.* ADHD=attention/deficit-hyperactivity disorder; ANEW = Lang Affective Norms for English Words; APD=antisocial personality disorder; AT=Adapted Task; BART = Balloon Analogue Risk Task; CBCL=Child Behavior Checklist; CBG=Colorado Balloon Game; CD=conduct disorder; CDT=Choice Delay Task; CI=Choice Impulsivity; CGT=Cambridge Gambling Task; CP=Clicking Paradigm; CPT=Continuous Performance Task; CT=Card Playing Task; DBD=disruptive behavior disorders; DBD-RS=Disruptive Behavior Disorders Rating Scale; DBPs=disruptive behavior problems; DD=Delay Discounting; DG=Delay of Gratification; DM=Decision Making; DOT = Door Opening Task; E=Emotional (Adapted Task); FPGT=Foregone Payoff Gambling Task; G=Gambling; GDT=Game of Dice Task; GMT=Gambling Machine Task; GT=Gamble Task; HDT=Hungry Donkey Task; IAPS=International Affective Picture System; IGT=Iowa Gambling Task; JPT=Jackpot Task; M=mean; MIDA = Maudsley Index of Delay Aversion; MMG=Make a Match Game; MRT =Mean Reaction Time; ODD=oppositional defiant disorder; PDT=Probabilistic Discounting Task; R=Rewarded (Adapted Task); RDT=Reward Dominance Task; RDMUR=Rational Decision Making Under Risk Task; RT=Reaction Time; RTV=Reaction Time Variability; SCP=Single Choice Paradigm; SDQ=Strengths and Difficulties Questionnaire; SMD=Standardized Mean Difference; SSRT=Stop Signal Reaction Time; SUD=Substance Use Disorder; TD=typically developing; TDP=Temporal Discounting Paradigm; WMST=Wisconsin Monster Sorting Test.
^a^See *Figure 1* for Conceptual Diagram of Domains, Subdomains, and Task Types reviewed in the present study.
^b^These reviews focused on the effects of reinforcement on inhibition; listed tasks were studied with/without added reinforcement, though specific reinforcers and schedules utilized differ by individual study.
^c^This study includes groups other than ADHD (and DBPs). The present review only considered results regarding ADHD (with/without DBPs) compared to TD.

^d^One study (Bitsakou et al., 2009) also investigated delay frustration and delay RT tasks.
^e^This study included both AT(R) and DM(G) tasks. The AT(R) findings are the primary focus as DM(G) is comprehensively reviewed elsewhere.
^f^This study used the language “disruptive, impulse-control, and conduct disorders (DICCD)”.
^g^Authors describe that the emotional words were tested and validated by a previous student dissertation.
